# *Lactobacillus casei* and Its Supplement Alleviate Stress-Induced Depression and Anxiety in Mice by the Regulation of BDNF Expression and NF-κB Activation

**DOI:** 10.3390/nu15112488

**Published:** 2023-05-26

**Authors:** Xiaoyang Ma, Yoon-Jung Shin, Hee-Seo Park, Ji-Woong Jeong, Joo Yun Kim, Jae-Jung Shim, Jung-Lyoul Lee, Dong-Hyun Kim

**Affiliations:** 1 Neurobiota Research Center, College of Pharmacy, Kyung Hee University, Seoul 02447, Republic of Korea; xiaoyangma12@gmail.com (X.M.); nayo971111@naver.com (Y.-J.S.); xlvksl1997@khu.ac.kr (H.-S.P.); 2 R&BD Department, hy Co., Ltd., Seoul 06530, Republic of Korea; woongshow@hy.co.kr (J.-W.J.); monera@hy.co.kr (J.Y.K.); jjshim@hy.co.kr (J.-J.S.); jlleesk@hy.co.kr (J.-L.L.); 3 PBLbiolab, Seoul 02823, Republic of Korea

**Keywords:** *Lactobacillus casei*, restraint stress, depression, anxiety, fecal microbiota

## Abstract

Stress-induced depression and anxiety (DA) are closely connected to gastrointestinal inflammation and dysbiosis, which can suppress brain-derived neurotrophic factor (BDNF) in the brain. Herein, we isolated the BDNF expression-inducing probiotics *Lactobacillus casei* HY2782 and *Bifidobacterium lactis* HY8002 in lipopolysaccharide-stimulated SH-SY5Y cells. Then, we investigated the effects of HY2782, HY8002, anti-inflammatory L-theanine, and their supplement (PfS, probiotics-fermented L-theanine-containing supplement) on DA in mice exposed to restraint stress (RS) or the fecal microbiota of patients with inflammatory bowel disease and depression (FMd). Oral administration of HY2782, HY8002, or L-theanine alleviated RS-induced DA-like behaviors. They also decreased RS-induced hippocampal interleukin (IL)-1β and IL-6 levels, as well as NF-κB-positive cell numbers, blood corticosterone level, and colonic IL-1β and IL-6 levels and NF-κB-positive cell numbers. L-theanine more potently suppressed DA-like behaviors and inflammation-related marker levels than probiotics. However, these probiotics more potently increased RS-suppressed hippocampal BDNF level and BDNF^+^NeuN^+^ cell numbers than L-theanine. Furthermore, HY2782 and HY8002 suppressed RS-increased *Proteobacteria* and *Verrucomicrobia* populations in gut microbiota. In particular, they increased *Lachnospiraceae* and *Lactobacillacease* populations, which are closely positively associated with hippocampal BDNF expression, and suppressed *Sutterellaceae*, *Helicobacteriaceae*, *Akkermansiaceae*, and *Enterobacteriaceae* populations, which are closely positively associated with hippocampal IL-1β expression. HY2782 and HY8002 potently alleviated FMd-induced DA-like behaviors and increased FMd-suppressed BDNF, serotonin levels, and BDNF-positive neuronal cell numbers in the brain. They alleviated blood corticosterone level and colonic IL-1β α and IL-6 levels. However, L-theanine weakly, but not significantly, alleviated FMd-induced DA-like behaviors and gut inflammation. BDNF expression-inducing probiotic (HY2782, HY8002, *Streptococcus thermophilus*, and *Lactobacillus acidophilus*)-fermented and anti-inflammatory L-theanine-containing supplement PfS alleviated DA-like behaviors, inflammation-related biomarker levels, and gut dysbiosis more than probiotics or L-theanine. Based on these findings, a combination of BDNF expression-inducing probiotics with anti-inflammatory L-theanine may additively or synergistically alleviate DA and gut dysbiosis by regulating gut microbiota-mediated inflammation and BDNF expression, thereby being beneficial for DA.

## 1. Introduction

Stress causes endocrine disruption, immune imbalance, including inflammation, and gut dysbiosis [1,2]. Stress-induced gut dysbiosis is associated with the overproduction of endotoxins such as gram-negative bacterial lipopolysaccharide (LPS), which can cause gut inflammation [3,4,5]. These endotoxins can be translocated to the brain through the blood, and suppress serotonin and brain-derived neurotropic factor (BDNF) levels in the hippocampus, resulting in psychiatric disorders including depression and anxiety (DA) through neuroinflammation [4,6,7].

Probiotics improve gut dysbiosis, metabolic diseases, immune imbalance, and neurodegenerative disorders [8,9,10,11]. *Bifidobacterium longum* NCC3001 alleviates depression and colitis in rodents [12]. Moreover, NCC3001 partially alleviates depression scores in patients with irritable bowel syndrome [13]. NVP1704, a *Lactobacillus reuteri* and *Bifidobacterium adolescentis* mix, improves restraint stress (RS)-related DA-like behaviors and sleep disturbances in volunteers with depression and insomnia [14]. NVP1704 also alleviates *Escherichia coli*-induced DA in mice [15]. *Bifidobacterium breve* CCFM1025 mitigates depression in rodents treated with chronic unpredictable mild stress (CUMS) by alleviating gut microbial abnormalities [16]. *Bifidobacterium breve* CCFM1025 reduces depressive scores in volunteers with major depression disorder by modulating gut microbiota and serotonin turnover [17]. *Lactobacillus rhamnosus* HN001 improves DA in CUMS-treated mice and pregnancy [18,19]. *Lactobacillus pentosus* NK357 also mitigates gut inflammation (GI) and gut dysbiosis (GD) in pathogen-exposed mice [20].

Some herbs, such as lavender, passionflower, saffron, and green tea have a well-known ability to alleviate DA [21,22]. Green tea contains polyphenols (especially epigallocatechin-3-gallate), polysaccharides, L-theanine, and caffeine [23]. In particular, L-theanine mitigates CUMS-induced depression in rats and DA-related symptoms in healthy volunteers [24]. However, the effects of supplements combined with probiotics and herbal constituents for the treatment of stress-induced DA have been not sufficiently studied.

Therefore, to understand the combined effects of BDNF expression-inducing probiotics and anti-inflammatory natural product constituent L-theanine on DA, we first isolated BNDF expression-inducing probiotics in LPS-stimulated SH-SY5Y cells, then investigated the effects of BDNF expression-inducing probiotics (*Lactobacillus casei* HY2782 and *Bifidobacterium lactis* HY8002), anti-inflammatory L-theanine, and their supplement on DA in RS-exposed or gut microbiota-transplanted mice.

## 2. Materials and Methods

### 2.1. Bacterial Strains

HY2782 and HY8002 were isolated from human fecal lactic acid bacteria collection, and *Streptococcus thermophilus* and *Lactobacillus acidophilus* strains were purchased from CSI (Culture System Inc., Mishawaka, IN, USA) as commercial strains for starter culture. Probiotics were cultured in MRS broth (BD, Franklin Lakes, NJ, USA) at 37 °C for 18 h, centrifuged (4000× *g*, 4 °C, 10 min), and washed with saline twice. The collected cells were resuspended in saline for in vivo experiments and heat (75 °C for 15 min)-tyndallized for in vitro experiments.

### 2.2. Preparation of Probiotics-Fermented Supplement (pFS)

For the preparation of PfS, *L. casei* (HY2782), *Bifidobacterium lactis* (HY8002), *Streptococcus thermophilus*, and *Lactobacillus acidophilus*, which were contained in the PROBIOTICS ABCT-3 starter culture (Culture System Inc., Mishawaka, IN, USA), were fermented in fresh milk, skim milk powder, dairy cream, and lactase (<0.002%, Maxilact LGi 5000, DSM Food Specialities, Libercourt, France); L-theanine (>98% pure synthetic L-theanine, Hunan Nutramax Inc., Hunan, Changsha, China) was added after the fermentation. In the final supplement, L-theanine content was 2 mg/mL, and total probiotic population was 2 × 10^8^ colony forming units (CFU)/mL.

### 2.3. SH-SY5Y Cell Culture and Selection of BDNF Expression-Inducing Probiotics

SH-SY5Y cells were cultured in DMEM containing 1% antibiotic-antimycotic and 5% FBS (37 °C, 95% air/5% CO_2_). For the BDNF expression assay, SH-SY5Y cells (1 × 10^6^ cells/mL) were incubated with LPS (100 ng/mL) in the absence or presence of probiotics (1 × 10^4^ or 1 × 10^6^ CFU/mL) for 24 h. BDNF levels were assayed using a commercial enzyme-linked immunosorbent assay (ELISA) kit.

### 2.4. Animals

C57BL/6 mice (male, 7 weeks old) were provided from Samtaco Inc. (Osan-shi, Seoul, Republic of Korea), maintained in a controlled room with water and food ad libitum, and acclimatized for 7 days before the use of experiment. All animal experiments were approved by the Committee for the Care and Use of Laboratory Animals in Kyung Hee University (IACUC No, KHUASP(SE)-23005) and were ethically carried out in accordance with the Guidelines of the University for Laboratory Animals Care and Use.

Mice with DA were prepared as previously reported [25]. (1) For the preparation of mice with RS-induced DA, mice were exposed to RS daily for 7 days. From the next day, test agents (Hy1, 2 × 10^8^ CFU/mouse of HY2782; Hy2, 2 × 10^8^ CFU/mouse of HY8002; Tn, 2 mg/kg of L-theanine; PfS, fermented supplement containing 2 × 10^8^ CFU/mouse of probiotics (consisted of *L. casei, B. lactis, S. thermophilus*, and *L. acidophilus*) and 2 mg/kg of L-theanine) were orally administered daily for 10 days. (2) For the preparation of mice with DA induced by fecal microbiota of patients with inflammatory bowel disease and depression (FMd), first FMd was cultured in general anaerobic medium (GAM) broth (Nissui Pharm. Co., Tokyo, Japan), as previously reported [25], centrifuged, and washed with saline twice. The collected FMd was orally transplanted into mice daily for 5 days. From the next day, test agents (Hy1, 2 × 10^8^ CFU/mouse of HY2782; Hy2, 2 × 10^8^ CFU/mouse of HY8002; Tn, 2.0 mg/kg of L-theanine; PfS, fermented supplement containing 2 × 10^8^ CFU/mouse of probiotics (consisted of *L. casei*, *B. lactis*, *S. thermophilus*, and *L. acidophilus*) and 2 mg/kg of L-theanine) were then orally administered daily for 10 days.

DA-like behaviors (one task in one day) were assessed from the next day after the final probiotic treatment. Mice were euthanized by exposure to CO_2_ in a chamber and then sacrificed by cervical dislocation. Sera, brains, colons, and feces were collected and stored at −80 °C for biomarker assays.

### 2.5. Behavioral Tasks

The open field test (OFT) was performed in a chamber (40 × 40 cm; center zone, 20 × 20 cm) equipped with a record camera for 10 min and quantified using the EthoVision XT software [26]. The elevated plus maze task (EPMT) and tail suspension test (TST) were assessed in the plus-maze apparatus and table edge, as previously reported [25].

### 2.6. Eenzyme-Linked Immunosorbent Assay (ELISA)

Hippocampus, hypothalamus, and colon tissues were lysed in a RIPA lysis buffer and centrifuged, as previously reported [4]. Sera were prepared as previously reported [20]. BDNF, IL-1β, IL-2, IL-6, and tumor necrosis factor (TNF)-α levels were determined using commercial ELISA kits (R&D Systems, Minneapolis, MN, USA) [27].

### 2.7. Immunofluorescence Staining

Mice were transcardially perfused with paraformaldehyde. Their hypothalamus and colon tissues were sectioned and incubated with primary antibodies for 12 h, then treated with secondary antibodies and observed using a confocal microscope, as previously reported [28]. The sections were incubated with primary antibodies against BDNF, NeuN, NF-κB, Iba1, and/or CD11c for 12 h, then treated with secondary antibodies conjugated with Alexa Fluor 594 or Alexa Fluor 488 (1:200, Invitrogen, Waltham, MA, USA) for 2 h, and observed using a confocal microscope.

### 2.8. Microbiota Analysis

Microbiota genomic DNA was extracted from the stool of mice using a QIAamp DNA stool mini kit. Next, 16S rRNA genes were amplified and sequenced, as previously reported [29]. Sequenced data were deposited in NCBI (PRJNA962265).

### 2.9. Statistics

Data are expressed as mean ± S.D. using GraphPad Prism 9. The significant differences were analyzed using one-way ANOVA followed by Duncan’s multiple range test (*p* < 0.05). The correlation between gut microbiota and BDNF expression or IL-1β level was analyzed using the Spearman correlation coefficient.

## 3. Results

### 3.1. L. casei and B. lactis Up-Regulated LPS-Suppressed BDNF Expression in SH-SY5Y Cells

**Figure 1:**
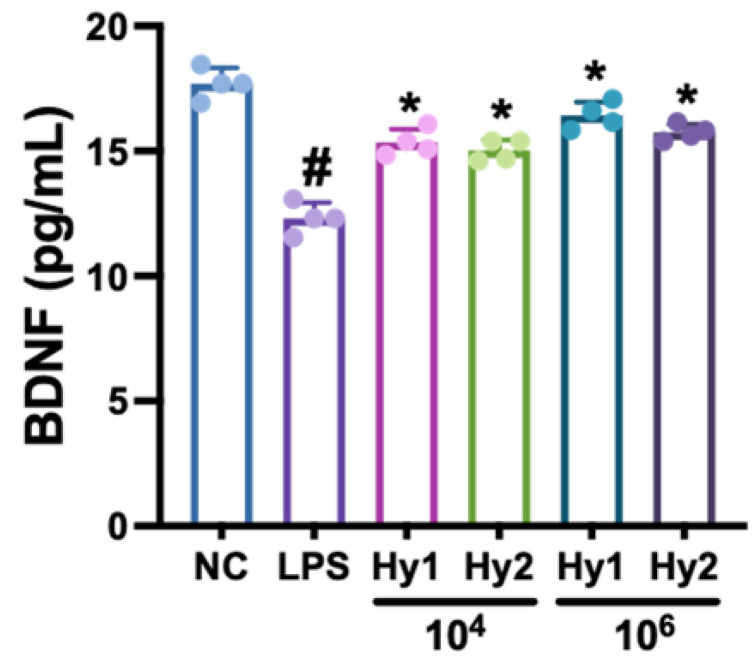
Effects of *L. casei* HY2782 and *B. lactis* HY8002 on LPS-suppressed BDNF expression in SH-SY5Y cells. Probiotics (Hy1, HY2782; Hy2, HY8002) were administered at a dose of 1 × 10^4^ or 1 × 10^6^ CFU/mL. LPS and NC mice were treated with LPS (100 ng/mL) or saline, respectively. Dn = 4. ^#^ *p* < 0.05 vs. NC. * *p* < 0.05 vs. LPS.

To select stress-induced DA-ameliorating probiotics, we screened BDNF expression-inducing probiotics from our lactic acid bacteria collection. Of the tested bacteria, *L. casei* HY2782 and *B. lactis* HY8002 potently increased LPS-suppressed BDNF expression in SH-SY5Y cells compared to that of the normal control (NC) (Figure 1).

### 3.2. L. casei, B. lactis, L-Theanine, and Their Supplement PfS Improved RS-Induced DA-like Behaviors and Neuroinflammation in Mice

**Figure 2:**
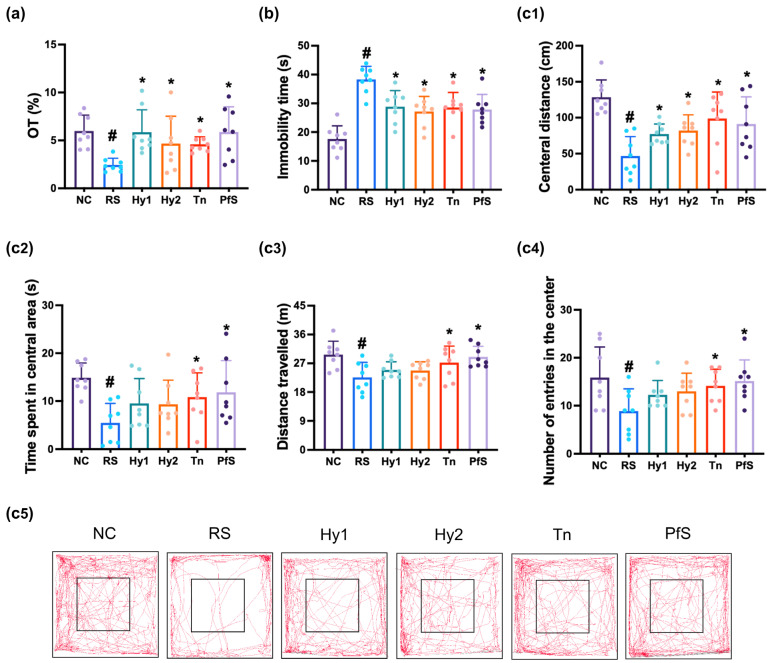
Effects of *L. casei* HY2782, *B. lactis* HY8002, L-theanine, and their supplement on RS-induced DA-like behaviors in mice. Behaviors were assessed in the EPMT (**a**), TST (**b**), and OPT (c: (**c1**), CD; (**c2**), CT; (**c3**), TN; (**c4**), CN; (**c5**), track path). Test agents (RS, saline; Hy1, HY2782; Hy2, HY8002; Tn, L-theanine; PfS, probiotics-fermented L-theanine-containing supplement) were orally gavaged. NC mice were treated with vehicle. n = 8. ^#^ *p* < 0.05 vs. NC. * *p* < 0.05 vs. RS.

**Figure 3:**
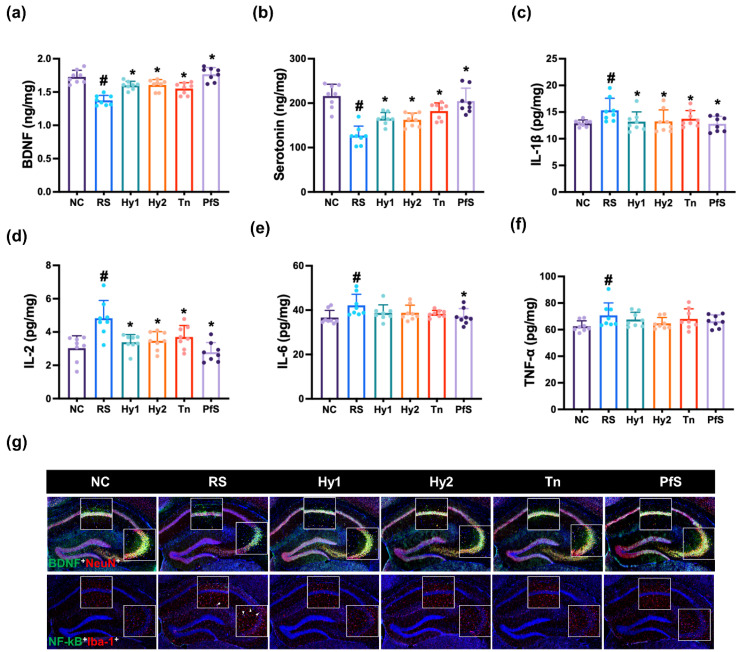
Effects of *L. casei* HY2782, *B. lactis* HY8002, L-theanine, and their supplement on RS-induced neuroinflammation in mice. Effects on hippocampal BDNF (**a**), serotonin (**b**), IL-1β (**c**), IL-2 (**d**), IL-6 (**e**), and TNF-α levels (**f**), and NF-κB^+^Iba1^+^ and BDNF^+^NeuN^+^ cell numbers (**g**). Test agents (RS, saline; Hy1, HY2782; Hy2, HY8002; Tn, L-theanine; PfS, probiotics-fermented L-theanine-containing supplement) were orally gavaged. NC mice were treated with vehicle. n = 8. ^#^ *p* < 0.05 vs. NC. * *p* < 0.05 vs. RS.

To study whether BDNF level-increasing probiotics could ameliorate DA in vivo, we investigated the effects of HY2782, HY8002, L-theanine, and their supplement PfS on RS-induced DA-like behaviors in mice (Figure 2). In NC mice, exposure to RS significantly decreased time in the open arm (OT) during the EPMT to 40.9% (F(6, 49) = 3.015, *p* < 0.014), reduced the central distance (CD), time spent in central area (CT), distance travelled (TD), and number of entries in the center (CN) during the OFT to 36.3% (F(6, 49) = 6.670, *p* < 0.001), 36.7% (F(6,4 9) = 2.925, *p* < 0.016), 76.1% (F(6, 49) = 3.478, *p* < 0.006), and 55.9% (F(6, 49) = 2.534, *p* < 0.032]), respectively, and expanded immobility time in the TST to 216.9% (F(6, 49) = 10.320, *p* < 0.001). However, HY2782, HY8002, and L-theanine increased RS-suppressed OT to 98.0%, 78.3%, and 76.9% (F(6, 49) = 3.015, *p* < 0.014), respectively, in NC mice. They also suppressed RS-increased immobility time to 163.6%, 154.1%, and 162.8% (F(6, 49) = 10.320, *p* < 0.001), respectively, and increased RS-suppressed CD to 60.1%, 63.9%, and 76.8% (F(6, 49) = 2.534, *p* < 0.032), respectively, in NC mice. Although L-theanine also potently alleviated RS-suppressed CT, TD, and CN, HY2782 and HY8002 weakly, but not significantly, increased them. They significantly decreased IL-1β and IL-2 levels in the hippocampus, while TNF-α levels were not affected (Figure 3). Although L-theanine significantly suppressed IL-6 expression, these probiotics did not significantly suppress it. They increased RS-suppressed serotonin, BDNF levels, and BDNF^+^NeuN^+^ cell population.

Treatment with PfS alleviated RS-induced DA-like behaviors in mice (Figure 2). PfS increased RS-suppressed OT to 98.5% (F(6, 49) = 3.015, *p* < 0.014), CD to 71.0% (F(6, 49) = 6.670, *p* < 0.001), and CT to 79.4% (F(6, 49) = 2.925, *p* < 0.016), in NC mice. PfS suppressed RS-exposed immobility time to 157.9% (F(6, 49) = 2.534, *p* < 0.032) in NC mice. Furthermore, PfS increased RS-suppressed serotonin, BDNF levels, and BDNF^+^NeuN^+^ cell numbers in the hippocampus, while RS-induced IL-1β and IL-6 levels and NF-κB^+^Iba1^+^ cell numbers decreased (Figure 3). PfS alleviated DA-like behaviors and neuroinflammation more potently than probiotics or L-theanine alone.

**Figure 4:**
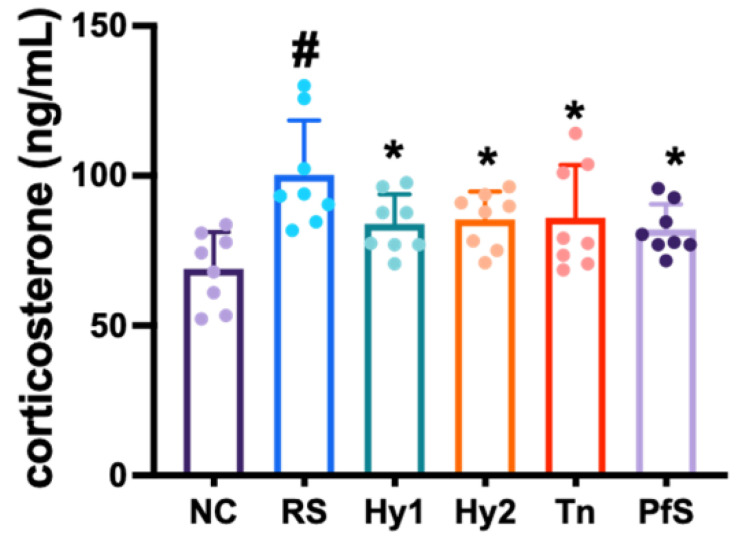
Effects of *L. casei* HY2782, *B. lactis* HY8002, L-theanine, and their supplement on corticosterone level in RS-exposed mice. Test agents (RS, saline; Hy1, HY2782; Hy2, HY8002; Tn, L-theanine; PfS, probiotics-fermented L-theanine-containing supplement) were orally gavaged. NC mice were treated with vehicle. n = 8. ^#^ *p* < 0.05 vs. NC. * *p* < 0.05 vs. RS.

Exposure to RS increased corticosterone levels in the blood (Figure 4). However, HY2782, HY8002, L-theanine, and PfS lowered RS-induced corticosterone levels.

### 3.3. L. casei, B. lactis, L-Theanine, and Their Supplement Improved RS-Induced GI and GD in Mice

**Figure 5:**
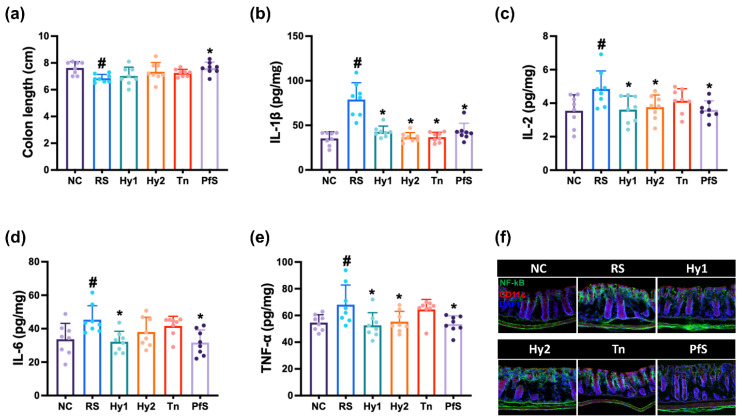
Effects of *L. casei* HY2782, *B. lactis* HY8002, L-theanine, and their supplement on RS-induced GI in mice. Effects on colon length (**a**), IL-1β (**b**), IL-2 (**c**), IL-6 (**d**), and TNF-α (**e**) levels, and NF-κB^+^CD11c^+^ cell numbers (**f**). Test agents (RS, saline; Hy1, HY2782; Hy2, HY8002; Tn, L-theanine; PfS, probiotics-fermented L-theanine-containing supplement) were orally administered. NC mice were treated with saline. n = 8. ^#^ *p* < 0.05 vs. NC. * *p* < 0.05 vs. RS.

The effects of HY2782 and HY8002 on RS-induced GI were investigated in the colon of mice (Figure 5). Exposure to RS induced colon shortening and increased IL-1β, IL-2, IL-6, and TNF-α levels, as well as NF-κB-positive cell numbers. However, HY2782 and HY8002 decreased RS-increased IL-1β and IL-6 levels, and NF-κB-positive cell numbers. L-theanine did not affect IL-1β, IL-2, IL-6, or TNF-α levels. PfS strongly suppressed IL-1β, IL-6, and TNF-α levels, as well as NF-κB-positive cell numbers, compared to those of L-theanine or probiotics.

**Figure 6:**
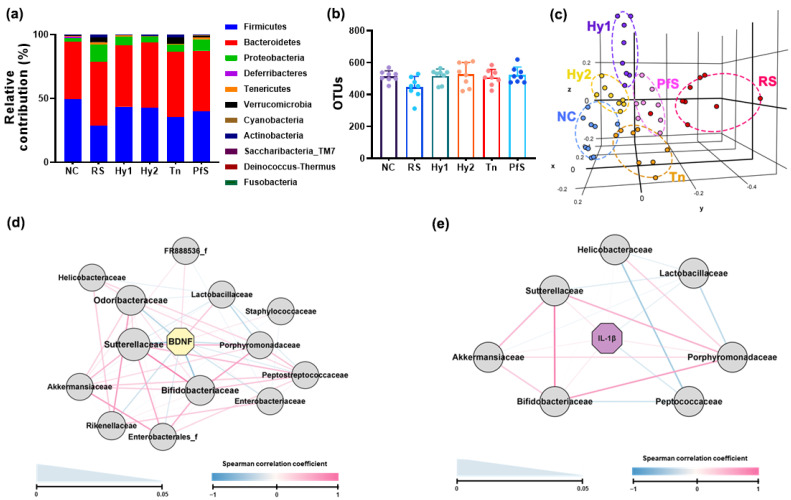
Effects of *L. casei* HY2782, *B. lactis* HY8002, L-theanine, and their supplement on RS-induced fecal microbiota alteration in mice. Effects on gut microbiota composition: (**a**), phylum level; (**b**), OTUs (α-diversity); (**c**), β-diversity (PCoA plot based on Jensen-Shannon analysis). The interrelation between gut microbiota and BDNF ((**d**), Spearman correlation coefficient) or IL-1β expression level ((**e**), spearman correlation coefficient). Test agents (RS, saline; Hy1, HY2782; Hy2, HY8002; Tn, L-theanine; PfS, probiotics-fermented L-theanine-containing supplement) were orally gavaged. n = 8.

The effects of probiotics, L-theanine, and their supplement on RS-induced gut microbiota alteration were investigated in mice (Figure 6, Supplementary Materials, Tables S1–S3). Exposure to RS-altered fecal microbiota composition: Exposure weakly, but not significantly, decreased α-diversity (OTUs) and shifted β-diversity (PCoA). RS increased *Proteobacteria*, *Verrucomicrobia*, and *Actinobacteria* populations, and decreased *Firmicutes* and *Deferribacteres* populations. However, HY2783, HY8002, and PfS suppressed RS-increased *Proteobacteria* and *Verrucomicrobia* populations and increased *Firmicutes* and *Deferribacteres* populations. Although L-theanine weakly, but not significantly, increased RS-suppressed *Firmicutes* population, it did not significantly affect RS-increased *Verrucomicrobia* or *Actinobacteria* populations. Of the gut microbiota, RS-decreased *Ruminococcaceae* and *Lactobacillacease* populations showed a positive correlation with BDNF expression levels, while RS-increased *Sutterellaceae*, *Helicobacteriaceae*, *Akkermansiaceae*, *Enterobacteriaceae*, and *Bifidobacteriaceae* numbers had a negative correlation. IL-1β expression levels showed a positive correlation with *Sutterellaceae*, *Bifidobacteiralceae*, and *Helicobacteriaceae* populations, while *Lactobacillaceae* and *Peptococcaceae* populations had a negative correlation.

### 3.4. L. casei, B. lactis, and Its Supplement Improved FMd-Induced DA-like Behaviors and Neuroinflammation in Mice

**Figure 7:**
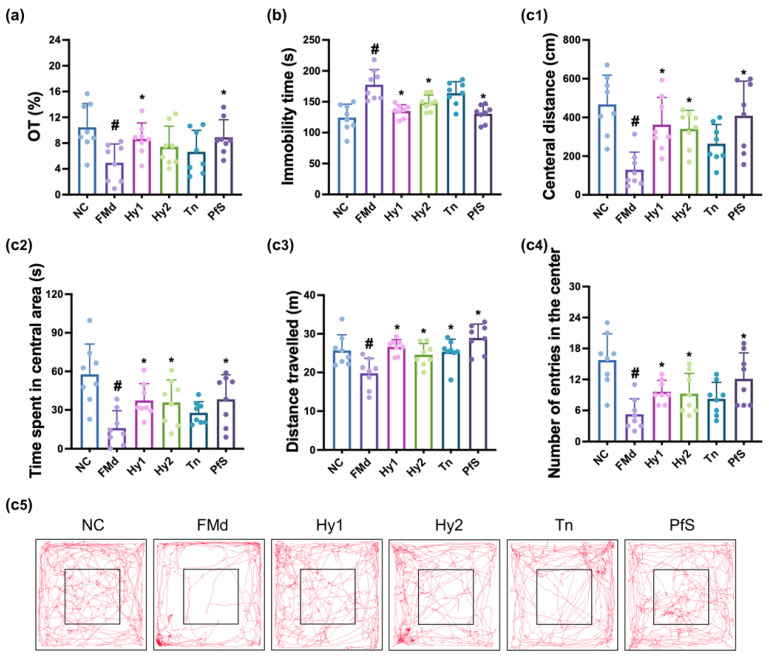
Effects of *L. casei* HY2782, *B. lactis* HY8002, L-theanine, and their supplement on FMd-induced DA-like behaviors in mice. Behaviors were assessed in the EPMT (**a**), TST (**b**), and OPT (c: (**c1**), CD; (**c2**), CT; (**c3**), TN; (**c4**), CN); (**c5**), track path). Test agents (FMd, saline; Hy1, HY2782; Hy2, HY8002; Tn, L-theanine; PfS, probiotics-fermented L-theanine-containing supplement) were orally gavaged. NC mice were treated with vehicle (saline) instead of test agents. n = 8. ^#^ *p* < 0.05 vs. NC. * *p* < 0.05 vs. FMd.

**Figure 8:**
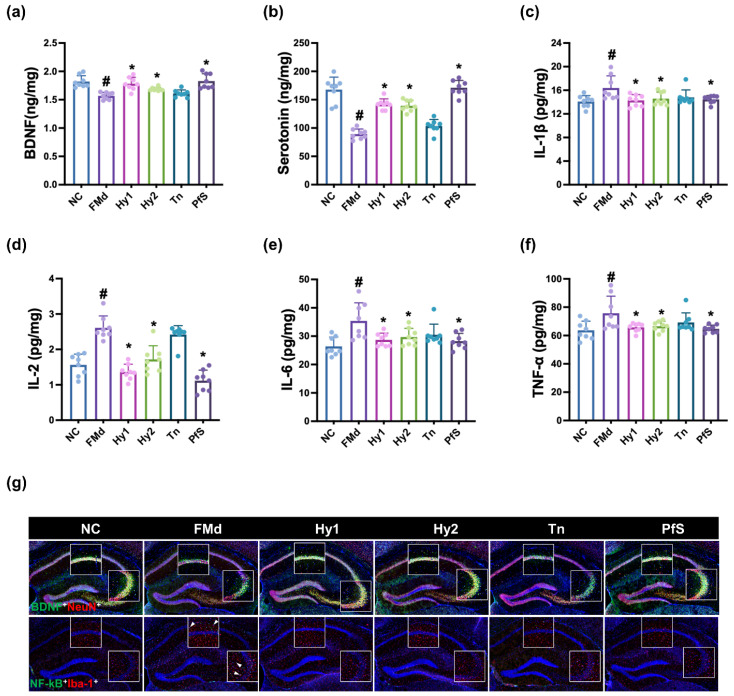
Effects of *L. casei* HY2782, *B. lactis* HY8002, L-theanine, and their supplement on FMd-induced neuroinflammation in mice. Effects on hippocampal BDNF (**a**), serotonin (**b**), IL-1β (**c**), IL-2 (**d**), IL-6 (**e**), and TNF-α levels (**f**), and NF-κB^+^Iba1^+^ and BDNF^+^NeuN^+^ cell numbers (**g**). Test agents (FMd, saline; Hy1, HY2782; Hy2, HY8002; Tn, L-theanine; PfS, probiotics-fermented L-theanine-containing supplement) were orally gavaged. NC mice were treated with vehicle (saline). n = 8. ^#^ *p* < 0.05 vs. NC. * *p* < 0.05 vs. FMd.

FMd transplantation causes DA with GI in mice [25]. To confirm the anti-depressive effects of HY2782, HY8002, and PfS, we examined their effects on FMd-induced DA in mice (Figure 7). In NC mice, FMd transplantation significantly decreased OT to 50.9% (F(5, 42) = 3.116, *p* < 0.018), reduced CD, CT, TD, and CN to 27.8% (F(5, 42) = 6.637, *p* < 0.001), 27.6% (F(5, 42) = 5.494, *p* < 0.001), 77.0% (F(5, 42) = 6.598, *p* < 0.001), and 33.3% (F(5, 42) = 6.756, *p* < 0.001), respectively, and increased immobility time to 142.8% (F(5, 42) = 11.000, *p* < 0.001). However, oral administration of HY2782 and HY8002 increased FMd-suppressed OT to 88.8% and 76.4% (F(5, 42) = 3.116, *p* < 0.018), respectively, in NC mice. They also decreased FMd-increased immobility time to 108.6% and 118.2% (F(5, 42) = 11.000, *p* < 0.001), respectively, and increased FMd-decreased CD to 78.0% and 72.9% (F(5, 42) = 6.637, *p* < 0.001), respectively, and CT to 64.7% and 62.1% (F(5, 42) = 5.494, *p* < 0.001), repectively, in NC mice. They significantly suppressed FMd-induced hippocampal TNF-α, IL-1β, IL-2, and IL-6 expression, while FMd-suppressed BDNF levels and BDNF^+^NeuN^+^ cell numbers increased (Figure 8). However, L-theanine did not significantly alleviate the DA-like behaviors of OT, immobility time, CD, or CT. Moreover, L-theanine weakly, but not significantly, suppressed FMd-induced proinflammatory cytokine expression in the hippocampus.

Treatment with PfS alleviated FMd-induced DA-like behaviors in mice. PfS increased FMd-suppressed OT to 86.5% (F(5, 42) = 3.116, *p* < 0.018), CD to 87.4% (F(5, 42) = 6.637, *p* < 0.001), and CT to 66.3% (F(5, 42) = 5.494, *p* < 0.001) in NC mice. Furthermore, PfS decreased FMd-increased immobility time to 105.0% (F(5, 42) = 11.000, *p* < 0.001) in NC mice. PfS also increased FMd-suppressed hippocampal serotonin, BDNF levels, and BDNF^+^NeuN^+^ cell numbers, while FMd-induced hippocampal TNF-α, IL-1β, and IL-6 levels, as well as NF-κB^+^Iba1^+^ cell numbers, decreased. PfS alleviated depression-like behaviors and neuroinflammation more potently than probiotics or L-theanine alone.

**Figure 9:**
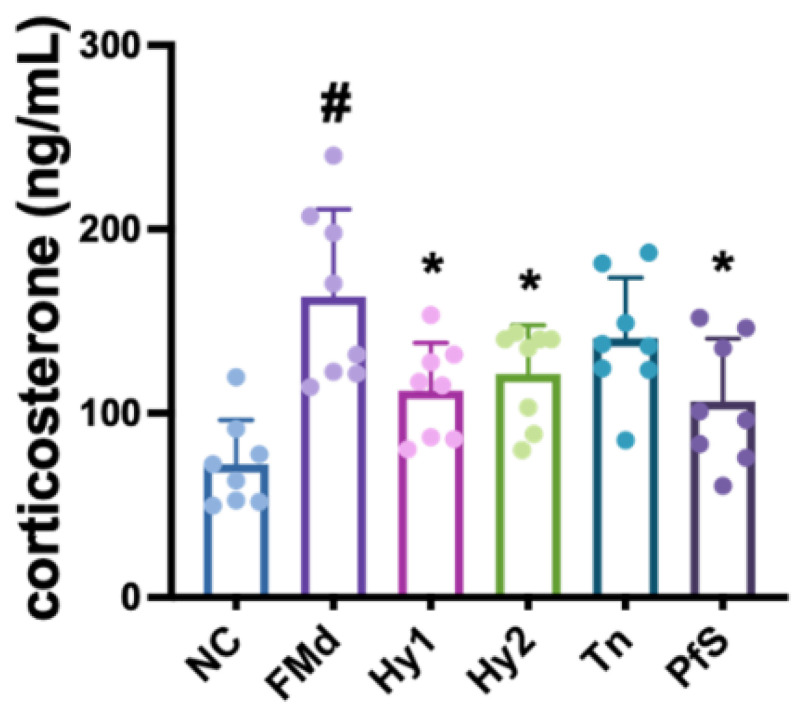
Effects of *L. casei* HY2782, *B. lactis* HY8002, L-theanine, and their supplement on corticosterone level in RS-exposed mice. Test agents (FMd, saline; Hy1, HY2782; Hy2, HY8002; Tn, L-theanine; PfS, probiotics-fermented L-theanine-containing supplement) were orally gavaged. NC mice were treated with vehicle. n = 8. ^#^ *p* < 0.05 vs. NC. * *p* < 0.05 vs. FMd.

FMd transplantation increased corticosterone levels in the blood (Figure 9). However, HY2782, HY8002, L-theanine, and PfS all lowered FMd-induced corticosterone levels.

### 3.5. L. casei, B. lactis, L-Theanine, and Their Supplement Improved FMd Transplantation-Induced GI in Mice

**Figure 10:**
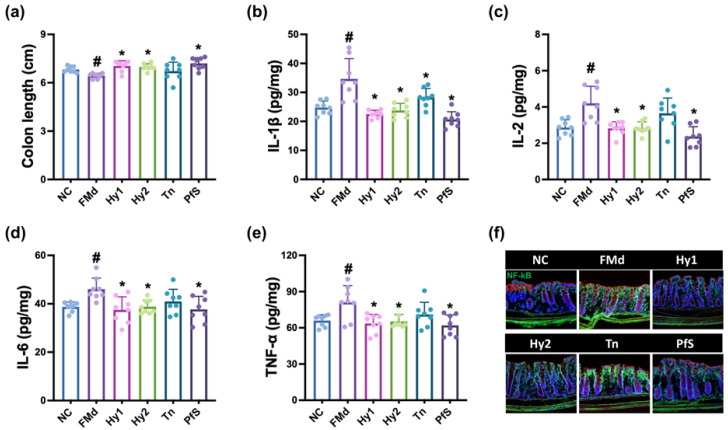
Effects of *L. casei* HY2782, *B. lactis* HY8002, L-theanine, and their supplement on FMd-induced GI in the colon of mice. Effects on colon length (**a**), IL-1β (**b**), IL-2 (**c**), IL-6 (**d**), and TNF-α (**e**) levels, and NF-κB^+^CD11c^+^ cell number (**f**). Test agents (FMd, saline; Hy1, HY2782; Hy2, HY8002; Tn, L-theanine; PfS, probiotics-fermented L-theanine-containing supplement) were orally gavaged. Normal control (NC) was treated with saline. n = 8. ^#^ *p* < 0.05 vs. NC. * *p* < 0.05 vs. FMd.

The effects of HY2782 and HY8002 on FMd-induced GI were investigated in the colon of mice (Figure 10). FMd transplantation increased IL-1β, IL-2, IL-6, and TNF-α levels, as well as NF-κB-positive cell numbers. However, HY2782 and HY8002 decreased IL-1β, IL-6, and TNF-α levels, as well as NF-κB-positive cell populations. However, L-theanine weakly suppressed these compared to probiotics. Treatment with PfS strongly suppressed the above levels compared to L-theanine or probiotics alone.

## 4. Discussion

Exposure to stressors induces the secretion of adrenocorticotropic hormone from the pituitary gland, which the stimulates the excretion of cortisol (corticosterone) from the adrenal gland through the hypothalamic–pituitary–adrenal axis and activates NF-κB signaling [30,31], while serotonin and BDNF levels decrease in the central nervous system and gastrointestinal tract, resulting in DA with neuroinflammation and colitis [32,33]. In the present study, exposure to RS increased blood corticosterone and IL-6 levels, and decreased hippocampal BDNF and serotonin levels, as well as BDNF-positive neuron cells. Moreover, exposure to RS increased IL-1β levels and NF-κB-positive cells in the brain and colon. RS increased DA-like behaviors. Furthermore, RS increased the gut *Proteobacteria* and *Verrucomicrobia* populations. Hippocampal BDNF expression levels were positively correlated with *Ruminococcaceae* and *Lactobacillacease* populations, which were negatively correlated with the expression levels of inflammatory cytokines such as IL-1β. Hippocampal BDNF expression levels were negatively correlated with *Sutterellaceae*, Helicobacteri-aceae, *Akkermansiaceae*, and *Enterobacteriaceae* populations, which were positively correlated with the expression levels of inflammatory cytokines such as IL-1β. Peirce and Alvina suggested that RS-induced depression could cause GI and GD [34]. Jang et al. reported that RS increased blood and fecal LPS levels through GD [3]. These observations suggest that RS may cause GI and DA by suppressing corticosterone-mediated BDNF and serotonin expression, and inducing corticosterone-mediated NF-κB signaling through LPS-overexpressed GD.

Here, we selected BDNF expression-increasing probiotics *L casei* HY2782 and *B. lactis* HY8002 in LPS-treated SH-SY5Y cells. These probiotics increased RS-suppressed BDNF and serotonin levels in the brain. However, they weakly suppressed RS-induced hippocampal and colonic proinflammatory cytokine and NF-κB-positive cell levels. Moreover, they weakly reduced blood corticosterone levels, and weakly alleviated RS-induced DA-like behaviors. Nevertheless, they shifted RS-fluctuated gut microbiota composition to that of NC. In particular, these probiotics suppressed RS-increased *Proteobacteria* and *Verrucomicrobia* populations. Furthermore, they increased Lachnospiraceae and *Lactobacillacease* populations, which are closely positively associated with hippocampal BDNF expression, and suppressed *Sutterellaceae*, *Helicobacteriaceae*, *Akkermansiaceae*, and *Enterobacteriaceae* populations, which are closely positively associated with hippocampal IL-1β expression. L-theanine also increased serotonin and BDNF levels, similarly to HY2782. However, L-theanine showed an anti-depressive effect. These results suggest that L-theanine may express its anti-depressive effects by regulating depressive factors such as neuropeptide Y and adrenaline [35], differently from probiotics.

Interestingly, HY2782 and HY8002 potently alleviated FMd-increased DA-like behaviors. They decreased FMd-induced hippocampal and colonic proinflammatory cytokine expression, and increased FMd-suppressed hippocampal BDNF expression and BDNF-positive neuron cell numbers. L-theanine strongly alleviated RS-induced DA-like behaviors associated with neuroinflammation and colitis. Its effects were more potent than those of probiotics in mice with RS-induced DA. However, L-theanine treatment hardly affected RS-fluctuated gut microbiota or hippocampal BDNF level. L-theanine hardly induced LPS-suppressed BDNF expression in vitro (Supplementary Materials, Figure S1). However, L-theanine weakly alleviated FMd-induced DA-like behaviors. L-theanine also weakly decreased the expression of IL-1β, IL-6, and TNF-α, which are neuroinflammation- and colitis-related biomarkers [36,37]. Zhang et al. found that L-theanine suppressed colitis in rodents by suppressing NF-κB signaling [38]. Gut inflammation is closely associated with the outbreak of psychiatric disorders, including DA and gut dysbiosis [4,39,40]. DA causes gut dysbiosis [41], which alters gut microbiota composition and overexpresses microbiota toxins such as LPS, and inflammation [25], which further induces DA [42]. Proinflammatory cytokines suppress BDNF and serotonin production in neuron cells [43]. The suppression of systemic inflammation alleviates DA in vivo [25]. We found that RS and FMd transplantation increased hippocampal and colonic IL-1β and IL-6 levels, as well as NF-κB-positive cell numbers, while hippocampal serotonin and BDNF levels decreased, as previously reported [25]. However, FMd transplantation more potently increased colonic IL-1β and IL-6 levels, as well as blood corticosterone levels, in mice that had been exposed to RS. These observations imply that BDNF expression-inducing probiotics, in particular *L. casei*, may alleviate RS-induced DA with gut dysbiosis through the modulation of gut microbiota composition and BDNF expression, while L-theanine may alleviate DA with neuroinflammation and colitis through the suppression of NF-κB activation.

Treatment with PfS, which is a probiotics-fermented L-theanine-containing supplement, alleviated RS- or FMd-induced DA-like behaviors, decreased hippocampal IL-1β and IL-6 levels, and increased RS- or FMd-suppressed hippocampal BDNF, serotonin levels, and BDNF-positive neuron cell numbers more potently than probiotics or L-theanine alone. Treatment also suppressed RS- or FMd-increased blood corticosterone levels, colonic IL-1β and IL-6 levels, and NF-κB-positive cell numbers more than probiotics or L-theanine alone. Furthermore, PfS suppressed *Proteobacteria* and *Verrucomicrobia* populations in RS-exposed mice. PfS also increased *Lactobacillacease* populations, which is closely positively associated with hippocampal BDNF expression, and suppressed *Sutterellaceae*, *Helicobacteriaceae*, *Akkermansiaceae*, and *Enterobacteriaceae* populations, which are closely positively associated with hippocampal IL-1β expression.

These observations suggest that the combination of BDNF expression-inducing probiotics, including HY2782 and HY8002, with anti-inflammatory L-theanine may additively or synergistically alleviate stress-induced DA by modulating gut microbiota-mediated inflammation and BDNF expression.

## 5. Conclusions

BDNF expression-inducing *L. casei* HY2782 and *B. lactis* HY8002 alleviated RD- or fecal microbiota-induced DA-like behaviors, neuroinflammation, and GI with GD by suppressing gut microbiota-mediated NF-κB activation and inducing BDNF expression. L-theanine alleviated RS-induced DA-like behaviors by suppressing NF-κB signaling. The combination of BDNF expression-inducing probiotics, including HY2782 and HY8002, with anti-inflammatory L-theanine may additively alleviate DA by modulating gut microbiota-mediated inflammation and BDNF expression, thereby being beneficial for DA.

## Data Availability

The data analyzed during the present study are available from the corresponding author.

## Supplementary Materials

The following supporting information can be downloaded at: https://www.mdpi.com/article/10.3390/nu15112488/s1, Table S1. Effects of HY2782 and its supplements on the fecal microbiota composition (at the phylum level). Table S2. Effects of HY2782 and its supplements on the fecal microbiota composition (at the family level). Table S3. Effects of HY2782 and its supplements on the fecal microbiota composition (at the genus level). Figure S1. Effect of L-theanine on LPS-suppressed BDNF expression in SH-SY5Y cells.

## Abbreviations

BDNF, brain-derived neurotropic factor; CD, central distance; CN number of entries in the center; CT, central area-spent time; CUMS, chronic unpredictable mild stress; DA, depression and anxiety; ELISA, enzyme-linked immunosorbent assay; EPMT, elevated plus maze task; GAM, general anaerobic medium; LPS, lipopolysaccharide; OFT, open field test; NC, normal control; SD, standard deviation; TD, distance travelled; TST, tail suspension test.
