# Supplementary material for: Lactobacillus casei and Its Supplement Alleviate Stress-Induced Depression and Anxiety in Mice by the Regulation of BDNF Expression and NF-κB Activation
Source: Nutrients. 2023 May 26;15(11):2488. doi: 10.3390/nu15112488 (PMC10255528; doi:10.3390/nu15112488)
Supplement: Supplementary file 1 [file nutrients-15-02488-s001.zip › nutrients-2395559-supplementary.pdf]

[Supplement]

***Lactobacillus casei* and Its Supplement Alleviate Stress-Induced Depression and Anx iety in Mice by the Regulation of BDNF Expression and NF-κB Activation**

Table S1. Effects of HY2782 and its supplements on the fecal microbiota composition (at the phylum level)

Taxon	Composition (%)					
	NC	RS	Hy1	Hy2	Tn	PfS
Firmicutes	49.4±10.6	28.4±7.1	43.5±9.2	42.8±5.9	35.2±7.9	40.0±8.2
Bacteroidetes	45.1±10.7	50.4±9.2	48.1±7.7	50.8±4.7	51.3±6.2	47.4±9.1
Proteobacteria	2.6±1.1	13.2±5.4	6.6±3.6	4.5±1.5	5.7±1.2	8.8±3.1
Deferribacteres	1.2±1.2	0.0±0.1	0.4±0.5	0.2±0.3	0.2±0.2	0.1±0.2
Tenericutes	0.6±0.3	2.0±0.7	0.8±0.4	0.2±0.1	0.2±0.2	1.6±0.7
Verrucomicrobia	0.5±0.6	3.6±3.4	0.2±0.3	0.1±0.0	5.0±2.2	1.3±1.9
Cyanobacteria	0.4±0.2	0.3±0.2	0.2±0.2	0.2±0.1	0.4±0.4	0.5±0.2
Actinobacteria	0.3±0.2	2.1±1.1	0.3±0.2	1.2±0.8	2.0±1.2	0.3±0.1
Saccharibacteria_TM7	0.0±0.0	0.0±0.0	0.0±0.0	0.0±0.0	0.0±0.0	0.0±0.0

Data indicate mean±SD.

Table S2. Effects of HY2782 and its supplements on the fecal microbiota composition (at the family level)

Taxon	Composition (%)					
	NC	RS	Hy1	Hy2	Tn	PfS
Muribaculaceae	35.7±7.0	29.5±11.7	30.7±6.0	41.9±4.9	37.4±6.8	36.3±6.2
Lachnospiraceae	29.5±8.7	18.5±5.9	28.8±7.1	24.5±8.4	21.6±8.1	25.4±8.6
Ruminococcaceae	10.5±3.4	4.8±3.7	7.1±2.6	5.2±2.3	4.9±1.3	5.4±2.3
Lactobacillaceae	7.2±2.7	3.0±1.0	6.2±2.8	11.5±8.2	4.8±1.6	5.0±2.5
Bacteroidaceae	6.1±3.3	7.2±3.6	6.3±2.3	2.6±1.0	4.0±2.8	3.7±2.6
Prevotellaceae	2.5±1.7	3.5±3.0	6.3±3.3	4.6±2.0	5.4±1.4	4.0±3.3
Christensenellaceae	1.3±0.5	0.7±0.3	0.8±0.4	0.7±0.5	0.7±0.4	0.7±0.4
Deferribacteraceae	1.2±1.2	0.0±0.1	0.4±0.5	0.2±0.3	0.2±0.2	0.1±0.2
Desulfovibrionaceae	0.8±0.8	0.5±0.5	1.3±0.9	1.7±1.0	1.2±1.0	1.1±0.9
Rikenellaceae	0.6±0.5	4.1±1.3	3.7±1.7	1.4±0.6	2.4±1.7	1.9±1.1
Pseudomonadaceae	0.6±0.6	1.1±1.8	0.8±0.7	0.6±0.7	1.2±0.8	0.8±1.1
Rhodospirillaceae	0.5±0.7	0.4±0.8	0.1±0.0	0.0±0.0	0.2±0.2	0.2±0.2
Akkermansiaceae	0.5±0.6	3.6±3.4	0.2±0.3	0.1±0.0	5.0±2.2	1.3±1.9

Sutterellaceae	0.5±0.3	2.2±1.3	0.5±0.2	1.0±0.7	2.8±1.0	0.7±0.6
FR888536_f	0.4±0.2	0.3±0.2	0.2±0.2	0.2±0.1	0.4±0.4	0.5±0.2
Erysipelotrichaceae	0.3±0.2	0.6±0.7	0.2±0.1	0.7±0.3	2.9±1.1	3.1±3.5
PAC001057_f	0.3±0.2	0.0±0.0	0.1±0.1	0.0±0.0	0.1±0.1	0.0±0.0
PAC000197_f	0.2±0.2	0.0±0.0	0.1±0.0	0.0±0.0	0.1±0.1	0.1±0.1
Dehalobacterium_f	0.2±0.1	0.1±0.1	0.2±0.1	0.2±0.1	0.2±0.1	0.1±0.1
Clostridiaceae	0.2±0.3	0.1±0.2	0.0±0.0	0.0±0.0	0.0±0.0	0.0±0.0
Coriobacteriaceae	0.2±0.2	0.1±0.1	0.2±0.1	0.2±0.1	0.1±0.1	0.1±0.1
Helicobacteraceae	0.1±0.1	8.3±4.5	0.9±0.2	1.1±0.6	0.2±0.1	5.9±2.5
Mogibacterium_f	0.1±0.0	0.0±0.0	0.1±0.0	0.1±0.0	0.1±0.0	0.1±0.0
Odoribacteraceae	0.1±0.1	2.8±1.6	1.0±0.3	0.2±0.1	2.0±1.7	0.8±0.4
Bifidobacteriaceae	0.1±0.1	2.0±1.1	0.1±0.1	1.0±0.8	1.9±1.2	0.2±0.1

Data indicate mean±SD.

Table S3. Effects of HY2782 and its supplements on the fecal microbiota composition (at the genus level)

Taxon	Composition (%)					
	NC	RS	Hy1	Hy2	Tn	PfS
PAC001068_g	14.2±2.9	9.3±4.9	7.2±2.1	15.0±3.4	11.3±1.9	13.9±3.8
KE159538_g	9.7±2.9	5.5±5.3	10.1±3.7	6.7±2.8	7.0±5.6	9.1±5.2
Lactobacillus	7.2±2.7	3.0±1.0	6.2±2.8	11.4±8.1	4.8±1.6	5.0±2.5
Bacteroides	6.1±3.3	7.2±3.6	6.3±2.3	2.6±1.0	4.0±2.8	3.7±2.6
PAC000186_g	5.9±2.1	4.0±1.4	6.1±2.2	7.3±2.8	6.6±2.6	4.7±1.5
Pseudoflavonifractor	3.7±1.4	1.9±1.8	2.7±0.6	2.0±0.9	1.8±0.4	2.0±0.9
Oscillibacter	3.6±1.4	1.8±1.7	2.8±1.4	2.0±1.1	1.7±0.6	2.2±0.9
PAC000198_g	3.5±1.0	5.4±1.8	2.7±0.9	3.3±1.1	5.5±1.1	3.8±1.2
PAC001512_g	3.0±1.3	1.4±1.1	0.0±0.0	0.0±0.0	0.2±0.1	0.3±0.4
PAC002400_g	2.2±0.9	0.5±0.3	0.8±0.3	0.6±0.3	1.1±0.4	0.8±0.5
PAC001112_g	2.1±2.9	1.9±2.2	4.4±3.2	6.2±3.3	5.1±3.4	3.5±3.8
PAC000664_g	2.1±1.7	1.9±1.6	3.5±2.1	4.1±2.7	3.1±2.2	3.0±2.6
PAC001544_g	1.8±2.2	0.1±0.1	0.2±0.5	0.1±0.1	0.1±0.2	0.1±0.2
PAC001091_g	1.5±0.8	0.3±0.2	0.6±0.5	0.5±0.4	0.5±0.5	0.3±0.3
PAC001092_g	1.3±0.9	1.0±0.9	1.8±1.0	1.9±1.4	1.6±1.2	1.6±1.1
Eubacterium_g6	1.2±1.1	0.4±0.4	0.9±1.0	0.8±0.6	0.6±0.6	1.2±1.6
Mucispirillum	1.2±1.2	0.0±0.1	0.4±0.5	0.2±0.3	0.2±0.2	0.1±0.2
Prevotellaceae_uc	1.1±1.0	2.6±2.1	2.3±3.4	1.3±1.4	4.3±1.8	0.9±1.2
Anaerotignum	1.1±0.5	0.8±0.5	0.8±0.4	0.6±0.3	0.5±0.2	0.6±0.5
Muribaculum	1.1±0.7	1.9±0.8	2.4±0.6	2.2±0.8	1.7±0.8	2.3±0.8
KE159571_g	0.9±0.8	0.7±0.5	1.5±1.3	1.3±0.7	0.8±0.8	0.9±0.6
Paraprevotella	0.9±0.8	0.2±0.1	0.4±0.6	0.4±0.3	0.2±0.2	0.3±0.2

PAC000661_g	0.9±0.5	0.2±0.1	0.3±0.2	0.2±0.1	0.3±0.3	0.1±0.1
LLKB_g	0.9±0.7	1.4±1.0	1.0±0.4	1.0±0.7	1.1±0.4	1.4±1.0
PAC001360_g	0.8±0.3	0.4±0.3	0.5±0.3	0.5±0.3	0.4±0.2	0.4±0.4

Data indicate mean±SD.

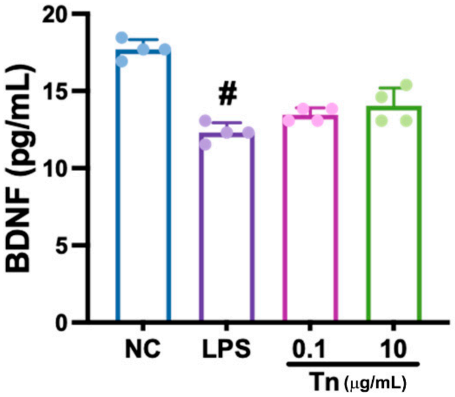

Figure S1. Effect of L-theanine on LPS-suppressed BDNF expression in SH-SY5Y cells. L-theanine was treated at a dose of 0.1 μg/mL or 10 μg/mL. LPS and NC were treated with LPS (100 ng/mL) and saline, respectively. n=4. # $p < 0.05$ vs. NC. * $p < 0.05$ vs. LPS.
